# Supplementary material for: Mitochondrial Dysfunction Causes Cell Death in Patients Affected by Fragile-X-Associated Disorders
Source: Int J Mol Sci. 2024 Mar 18;25(6):3421. doi: 10.3390/ijms25063421 (PMC10970216; doi:10.3390/ijms25063421)
Supplement: Supplementary file 1 [file ijms-25-03421-s001.zip › ijms-2861399-supplementary.pdf]

Supplementary Materials

Mitochondrial dysfunction causes cell death in patients affected by Fragile X-associated disorders

Martina Grandi[1], Chiara Galber[1,2], Cristina Gatto[1], Veronica Nobile[3], Cecilia Pucci[3], Ida Schaldemose Nielsen[1], Francesco Boldrin[4], Giovanni Neri[3], Pietro Chiurazzi[3,5], Giancarlo Solaini[1], Alessandra Baracca[1], Valentina Giorgio[1,2*], Elisabetta Tabolacci[3]

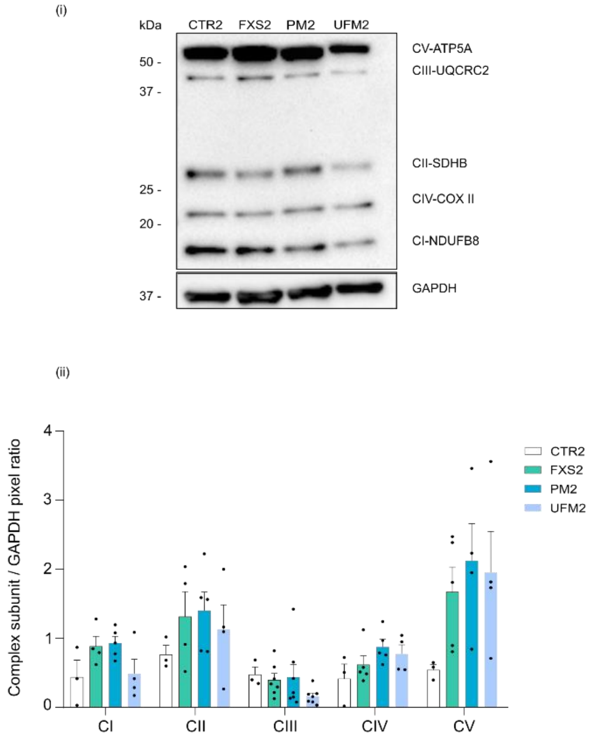

Figure S1. Mitochondrial oxidative phosphorylation in FXD fibroblasts. Western blotting is shown (i) of the indicated OXPHOS complex subunits and glyceraldehyde 3-phosphate dehydrogenase (GAPDH) as loading control in CTR2, FXS2, PM2 and UFM2 fibroblast lysates. The molecular marker is indicated on the left. Quantification (ii) is shown as band pixel ratio between each complex subunit and GAPDH (mean ± SEM of 4 independent experiments). Student's *t* test, NS on the control.

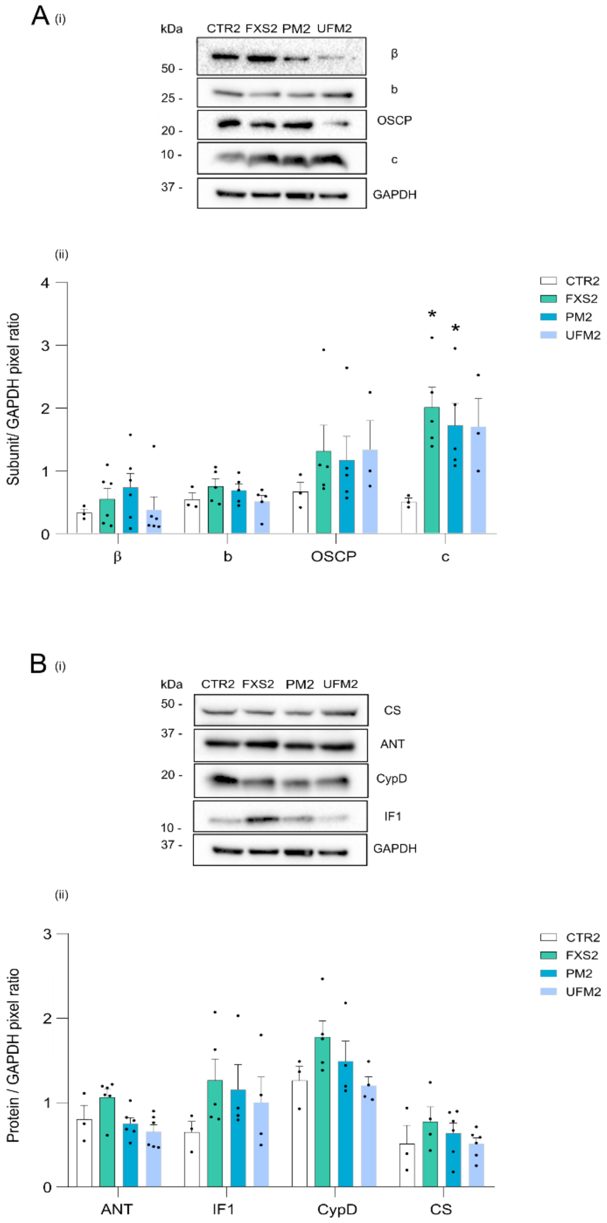

Figure S2. Quantification of mitochondrial proteins in FXD fibroblasts. In A, Western blotting (i) is shown of the ATP synthase β, b, OSCP and c subunits in CTR2, FXS2, PM2 and UFM2 cell lysates. Glyceraldehyde 3-phosphate dehydrogenase (GAPDH) is detected as loading control. Molecular markers are on the left. Mean pixel ratio (ii) ± SEM is shown between each ATP synthase subunit and GAPDH bands in CTR2, FXS2, PM2 and UFM2 fibroblast of at least 3 independent experiments. In B, Western blotting (i) is shown of citrate synthase (CS), adenine nucleotide translocator isoform 3 (ANT), cyclophilin D (CypD), IF1 (ATPase inhibitor) in CTR2, FXS2, PM2 and UFM2 cell lysates. Glyceraldehyde 3-phosphate dehydrogenase (GAPDH) is detected as loading control. The molecular marker is indicated on the left. Mean pixel ratio (ii) ± SEM is shown between each protein and GAPDH bands in CTR2, FXS2, PM2 and UFM2 fibroblast of at least 3 independent experiments. Student's *t* test, *p≤0.05 on the control.

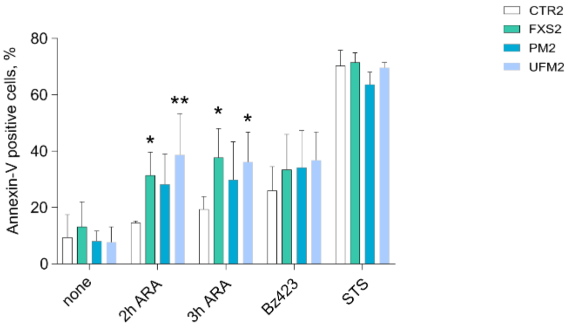

Figure S3. FXD fibroblasts are more sensitive to apoptosis than controls. Histogram shows the mean quantification ± SEM of apoptotic annexin V-positive cells (expressed as %) derived from CTR2, FXS2, PM2 and UFM2 patients. Treatment conditions are: 200 μM arachidonic acid (ARA) for 2 or 3 hours, 100 μM benzodiazepine 423 (Bz423) for 8 hours, or 2 μM staurosporine (STS) for 24 hours. Data are from at least 4 independent experiments. *Two-way* Anova, *$p \leq 0.05$, **$p = 0.0012$ on the control.
